# Lycorine ameliorates diabetic nephropathy by targeting RAGE and inhibiting the HMGB1/RAGE/NF-κB signaling axis

**DOI:** 10.1186/s13020-026-01469-y

**Published:** 2026-07-16

**Authors:** Zheng Xu, Xuejin Jin, Miao Yuan, Huanyan Tao, Yutong Wang, Jingnan Zhang, Guang Liang, Qian Zhou

**Affiliations:** 1 Heart Center, Department of Cardiovascular Medicine, Zhejiang Provincial People’s Hospital, Affiliated People’s Hospital, Hangzhou Medical College, Hangzhou, China; 2 Zhejiang TCM Key Laboratory of Pharmacology and Translational Research of Natural Products, School of Pharmaceutical Sciences, Hangzhou Medical College, Hangzhou, 310014 Zhejiang China; 3 Department of Pharmacy and Institute of Inflammation, Zhejiang Provincial People’s Hospital, Affiliated People’s Hospital, Hangzhou Medical College, Hangzhou, 310014 Zhejiang China

**Keywords:** Diabetic nephropathy, Lycorine, RAGE, HMGB1, NF-κB

## Abstract

**Background:**

Diabetic nephropathy (DN) is a major microvascular complication of diabetes and a leading cause of end-stage renal disease. Chronic inflammation plays a pivotal role in the pathogenesis of DN. *Lycorine* (LY), a complex tetracyclic pyrrolo*[de]*phenanthridine alkaloid derived from the *Amaryllidaceae* family, possesses notable anti-inflammatory activity, yet its therapeutic potential in DN remains insufficiently defined.

**Methods:**

We evaluated the renoprotective effects of LY both in vivo and in vitro. Streptozotocin (STZ)-induced diabetic mice were treated with LY, and renal function and histopathological alterations were assessed. In vitro, human renal tubular epithelial HK-2 cells were exposed to high glucose plus palmitic acid (HG + PA) with or without LY. RNA-seq analysis was performed to identify LY-regulated pathways. Molecular docking, surface plasmon resonance (SPR) assay, and cellular thermal shift assay (CETSA) were used to evaluate the interaction between LY and receptor for advanced glycation end products (RAGE). RAGE siRNA-mediated knockdown was further conducted to determine whether RAGE is required for the protective effects of LY. Activation of the HMGB1/RAGE/NF-κB signaling axis and associated inflammatory mediators was analyzed by Western blotting and RT-PCR.

**Results:**

LY markedly alleviated renal injury in STZ-induced diabetic mice, as evidenced by reduced albuminuria, improved renal function, and attenuated renal fibrosis, apoptosis, oxidative stress, and inflammation. Notably, LY did not significantly alter blood glucose levels or body weight, indicating that its renoprotective effect was independent of glycemic control. In HG + PA-treated HK-2 cells, LY significantly suppressed apotosis, oxidative stress, and inflammatory cytokine expression. Mechanistically, RNA-seq analysis identified AGE-RAGE and NF-κB signaling pathways as key pathways modulated by LY. Molecular docking, SPR, and CETSA confirmed that LY directly interacted with RAGE. Moreover, RAGE knockdown largely abolished the additional protective effects of LY, supporting RAGE as a critical molecular target. LY inhibited HMGB1/RAGE-mediated NF-κB activation, as reflected by reduced HMGB1, RAGE, p-p65, and p-IκBα levels.

**Conclusion:**

LY ameliorates diabetic nephropathy without affecting blood glucose levels by directly targeting RAGE and suppressing the HMGB1/RAGE/NF-κB signaling axis. These findings identify LY as a potential RAGE-targeting therapeutic candidate for inflammation-driven diabetic kidney injury.

**Supplementary Information:**

The online version contains supplementary material available at 10.1186/s13020-026-01469-y.

## Introduction

Diabetic nephropathy is one of the most common and severe microvascular complications of diabetes mellitus and remains the leading cause of end-stage renal disease worldwide [3, 7, 13]. With the global prevalence of diabetes continuing to rise, the clinical and socioeconomic burden of diabetic nephropathy is increasing accordingly, highlighting the pressing need for effective therapeutic interventions [2, 25]. While diabetic nephropathy was traditionally viewed as a consequence of metabolic dysregulation and hemodynamic abnormalities [5, 20], growing evidence now indicates that persistent low-grade inflammation plays a fundamental role in driving renal injury and disease progression in diabetes [15, 16].

The receptor for advanced glycation end products (RAGE) serves as a key molecular link between metabolic stress and inflammatory injury in the diabetic kidney [6, 22]. Upon activation by ligands such as high mobility group box 1 (HMGB1), RAGE triggers a potent pro-inflammatory signaling cascade, predominantly through the nuclear factor-kappa B (NF-κB) pathway [21, 26]. This signaling activation drives the transcription of pro-inflammatory cytokines, including tumor necrosis factor-alpha (TNF-α), interleukin-1β (IL-1β), and interleukin-6 (IL-6), which exacerbate leukocyte recruitment, tubular damage, and fibrotic remodeling [28]. Consequently, therapeutic strategies that disrupt the HMGB1/RAGE/NF-κB axis hold considerable promise for mitigation inflammation-driven progression of diabetic nephropathy.

*Lycorine* (LY), a complex tetracyclic pyrrolo*[de]*phenanthridine alkaloid derived from the *Amaryllidaceae* family, has attracted considerable attention due to its broad spectrum of biological activities, including anti-cancer, anti-viral, and prominent anti-inflammatory effects [4, 10, 23, 27]. Although LY has been reported to alleviate inflammation in several pathological conditions [1, 19], its potential renoprotective role in diabetic nephropathy—and, critically, the specific molecular targets through which it modulates inflammatory signaling—remains insufficiently defined.

In this study, we systematically evaluated the renoprotective effects of LY in streptozotocin (STZ)-induced diabetic mice and high glucose plus palmitic acid (HG + PA)-stimulated human renal tubular epithelial (HK-2) cells. By integrating RNA-seq analysis, molecular docking, surface plasmon resonance assay, and cellular thermal shift assay, we sought to identify the molecular target and signaling mechanism underlying LY-mediated renal protection. We focused on RAGE because transcriptomic analysis highlighted enrichment of the AGE-RAGE and NF-κB pathways, and further investigated whether LY suppresses the HMGB1/RAGE/NF-κB signaling axis and downstream inflammatory cytokine production. These findings provide mechanistic insight into the anti-inflammatory and renoprotective actions of LY and support its potential development as a targeted therapeutic candidate for diabetic nephropathy.

## Materials and methods

### Reagents

LY (purity ≥ 98%) and STZ were purchased from Chengdu Must Bio-Technology Co., Ltd. (Chengdu, China) and Sigma-Aldrich (St. Louis, MO, USA), respectively.

### Animal experiments

Male C57BL/6 mice (6–8 weeks old) were obtained from the Laboratory Animal Center of Hangzhou Medical College and housed under specific pathogen-free conditions. All animals were acclimatized in a standard vivarium with controlled temperature (24 ± 2 °C), humidity (55 ± 5%), and a 12-h light/dark cycle. Throughout the study, mice had ad libitum access to standard rodent chow and water. All experimental procedures were performed in accordance with protocols approved by the Institutional Animal Care and Use Committee of Hangzhou Medical College (Approval No. 202502028, Date: May 15, 2025).

Type 1 diabetes mellitus was induced by a single intraperitoneal injection of STZ. STZ was dissolved in 100 mM citrate buffer (pH 4.5) and administered at a dose of 100 mg/kg body weight. Control mice received an equal volume of citrate buffer alone. After the induction period, fasting blood glucose levels were measured from tail vein blood using a clinical glucometer. Mice exhibiting sustained hyperglycemia (≥ 12 mmol/L) were considered diabetic and included in the subsequent experiments. To further confirm the establishment of the diabetic nephropathy model, renal function-related parameters were measured in addition to blood glucose levels. Urine samples were collected using metabolic cages, and blood samples were obtained from mice after fasting. Urinary albumin (H127-1–2, Nanjing Jiancheng Bioengineering Institute, Nanjing, China), creatinine (C011-2–1, Nanjing Jiancheng Bioengineering Institute, Nanjing, China), and blood urea nitrogen (C013-1–1, Nanjing Jiancheng Bioengineering Institute, Nanjing, China) were detected using commercial assay kits according to the manufacturers’ instructions. The urinary albumin-to-creatinine ratio (UACR) was calculated as urinary albumin divided by urinary creatinine.

STZ-induced diabetic mice were randomly assigned to four treatment groups (n = 7 per group): a vehicle control group (0.5% sodium carboxymethyl cellulose), a low-dose lycorine group (5 mg/kg), a high-dose lycorine group (20 mg/kg), and irbesartan (IRB, 30 mg/kg) group. Lycorine and irbesartan were administered by oral gavage every other day for a total of 10 weeks. Control mice received the vehicle on the same schedule. Body weight and fasting blood glucose were monitored weekly throughout the experimental period. At 18 weeks post-induction, mice were euthanized following established guidelines, blood and kidney tissue samples were subsequently harvested for analysis. Key renal function parameters, including blood urea nitrogen, urinary creatinine, and albumin levels, were then measured.

### Histomorphology

Kidney tissues were fixed in 4% paraformaldehyde for 48 h at room temperature, followed by paraffin embedding. Sections (5 μm thick) were prepared and subjected to histopathological staining: Hematoxylin and Eosin for general morphology, 0.1% Sirius Red for collagen deposition, Masson's Trichrome for fibrosis, and 0.5% Periodic acid-Schiff for evaluating basement membrane and mesangial expansion. All stained sections were examined under a light microscope (Leica Microsystems, Germany) at 200 × magnification for pathological assessment.

### Immunohistochemical determination

Kidney tissue sections underwent deparaffinization and rehydration, followed by endogenous peroxidase quenching with 3% hydrogen peroxide. Non-specific binding was blocked using 5% bovine serum albumin. After overnight incubation with primary antibodies at 4 °C, sections were treated with Horseradish Peroxidase-conjugated secondary antibodies. Positive signals were developed with diaminobenzidine, counterstained with hematoxylin, dehydrated, and finally imaged under a light microscope. Quantitative analysis was performed on the digital images using ImageJ software.

### Immunofluorescence staining

Paraffin-embedded kidney sections were deparaffinized, rehydrated, subjected to antigen retrieval, and blocked with bovine serum albumin. The sections were then incubated overnight at 4 °C with primary antibodies against RAGE (AF5309, Affinity) and HMGB1 (6893S, CST), followed by incubation with species-appropriate fluorescent secondary antibodies. Nuclei were counterstained with DAPI. Images were captured using a fluorescence microscope.

### RNA extraction, qPCR, and RNA-Seq

Total RNA was extracted from both renal tissue samples and cultured cells using TRIzol™ reagent (Invitrogen, Carlsbad, CA, USA). The isolated RNA was then reverse transcribed into complementary DNA (cDNA) employing the Evo M-MLV Reverse Transcription Kit (Accurate Biology, Hunan, China). Subsequently, quantitative real-time PCR (qPCR) amplification was performed with SYBR Green Master Mix (Accurate Biology) on a Bio-Rad CFX96 Touch™ thermal cycler (Hercules, CA, USA). Each amplification run incorporated negative control reactions, and product specificity was confirmed by melt curve analysis. Transcript levels were quantified using the 2^−ΔΔCt^ method, with data normalized to the expression of the housekeeping gene *Gapdh*. A portion of the renal tissue was processed for RNA sequencing.

### Western blotting

Proteins were isolated from kidney tissue and cultured cells using RIPA lysis buffer containing a protease inhibitor cocktail. Following homogenization, lysates were centrifuged at 12,000 × g for 15 min at 4 °C to remove debris, and the supernatant was collected for further analysis. Protein samples were separated by electrophoresis on 10% SDS‒polyacrylamide gels and then transferred to polyvinylidene difluoride membranes (Millipore, Burlington, MA, USA) using a semi‑dry transfer system. The membranes were blocked in 5% non‑fat milk prepared in tris buffered saline with tween-20 for 1 h at room temperature. Next, they were incubated overnight at 4 °C with specific primary antibodies, washed, and then incubated with horseradish peroxidase‑conjugated secondary antibodies (diluted 1:5000) for 1 h at room temperature. Signal detection was performed with Clarity™ ECL substrate (Bio‑Rad, Hercules, CA, USA), and band intensities were quantified using ImageJ software. To ensure equal sample loading, protein expression levels were normalized to GAPDH.

### Total superoxide dismutase (SOD) and glutathione peroxidase (GSH-Px)

The oxidative stress status in kidney tissues and human tubular epithelial cells was assessed by quantifying total superoxide dismutase and glutathione peroxidase activities with commercial assay kits (Beyotime, Shanghai, China).

### Reactive oxygen species assay

The reactive oxygen species (ROS) levels were assessed with commercial assay kits: for HK-2 cells using kit S0033S (Beyotime), and for animal tissues using kit ROS.T-W96-N1620 (Shanghai Enzyme-linked Biotechnology Co., Ltd.).

### Lipid peroxidation MDA assay

Malondialdehyde (MDA) levels were measured with a commercial assay kit (S0131S, Beyotime).

### Molecular docking

The interactions of RAGE were modeled based on crystallographic coordinates retrieved from the RCSB Protein Data Bank. The small‑molecule compound LY (PubChem CID: 72,378) was first geometry‑optimized using OpenBabel 2.4.1 (OpenEye Scientific) with the Merck Molecular Force Field (MMFF94). Molecular docking was then performed using AutoDock 4.2.6 (Scripps Research Institute). The docking protocol applied a Lamarckian genetic algorithm with the following settings: population size of 250, 2.5 × 10⁷ energy evaluations, and a grid spacing of 0.375 Å centered on the RAGE active site. Resulting binding poses were ranked according to predicted Gibbs free energy (ΔG). Structural analysis and visualization were carried out in PyMOL 2.5 (Schrödinger LLC), while binding‑pocket surfaces were mapped using Maestro 12.8.

### Cellular thermal shift assay

Human tubular epithelial cells were washed and resuspended in phosphate buffered saline, followed by lysis via three cycles of rapid freezing in liquid nitrogen and thawing. After centrifugation of the lysates at 12,000 g for 10 min at 4 °C, the supernatant was collected and evenly split into two aliquots. These aliquots were then treated with either 1 μM LY or an equivalent volume of DMSO for 30 min at 25 °C. Subsequently, each treated sample was further subdivided into nine equal parts and subjected to heat treatment for 3 min at incrementally increasing temperatures (40, 43, 46, 49, 52, 55, 58, 61, and 64 °C). Following heat exposure, the samples were centrifuged again at 12,000 g for 10 min at 4 °C. The resulting supernatants were mixed with loading buffer and boiled. Finally, target protein levels were assessed by Western blotting analysis.

### Surface plasmon resonance binding assay

The direct interaction between LY and RAGE was evaluated by surface plasmon resonance (SPR) using a Biacore T200 system (Cytiva, Marlborough, MA, USA) at 25 °C. Recombinant RAGE protein was covalently immobilized onto a CM7 sensor chip through standard amine-coupling chemistry in 10 mM sodium acetate buffer until an appropriate immobilization level was reached. A blank flow cell processed under the same activation and blocking conditions but without protein immobilization was used as the reference channel. LY was prepared as a series of concentrations and injected over the RAGE-coated surface at a flow rate of 30 μl/min, with association and dissociation monitored for 120 s and 180 s, respectively. After each binding cycle, the sensor surface was regenerated using 10 mM glycine–HCl (pH 2.5). The resulting sensorgrams were reference-subtracted and analyzed using Biacore Evaluation Software. The equilibrium dissociation constant (K_D_) was determined by fitting the steady-state binding responses to an affinity model.

### RNA interference

RAGE-specific gene silencing was achieved using custom-designed siRNA duplexes (GenePharma, Shanghai, China). Transfection complexes were prepared by incubating 50 µM siRNA with JetPRIMER® transfection reagent at room temperature for 10 min. These complexes were then delivered into human tubular epithelial cells under optimized transfection conditions. To assess silencing efficiency, RAGE protein levels were analyzed by immunoblotting 24 h post-transfection.

### Statistical analysis

Statistical analysis was performed using GraphPad Prism 9.0 (GraphPad Software Inc., San Diego, CA, USA). Data from three independent biological replicates are presented as mean ± standard deviation (SD). Differences among groups were evaluated by one-way ANOVA followed by Tukey's post hoc test for multiple comparisons. A *p*-value below 0.05 was considered statistically significant, and all significant findings were confirmed through permutation testing. Significance markers are denoted as follows: ^*^p < 0.05, ^**^p < 0.01, ^***^p < 0.001 *versus* the Control group; ^#^*p* < 0.05, ^##^*p* < 0.01, ^###^*p* < 0.001 *versus* the STZ group.

## Results

### LY ameliorates renal injury in STZ-induced diabetic mice

**Fig. 1:**
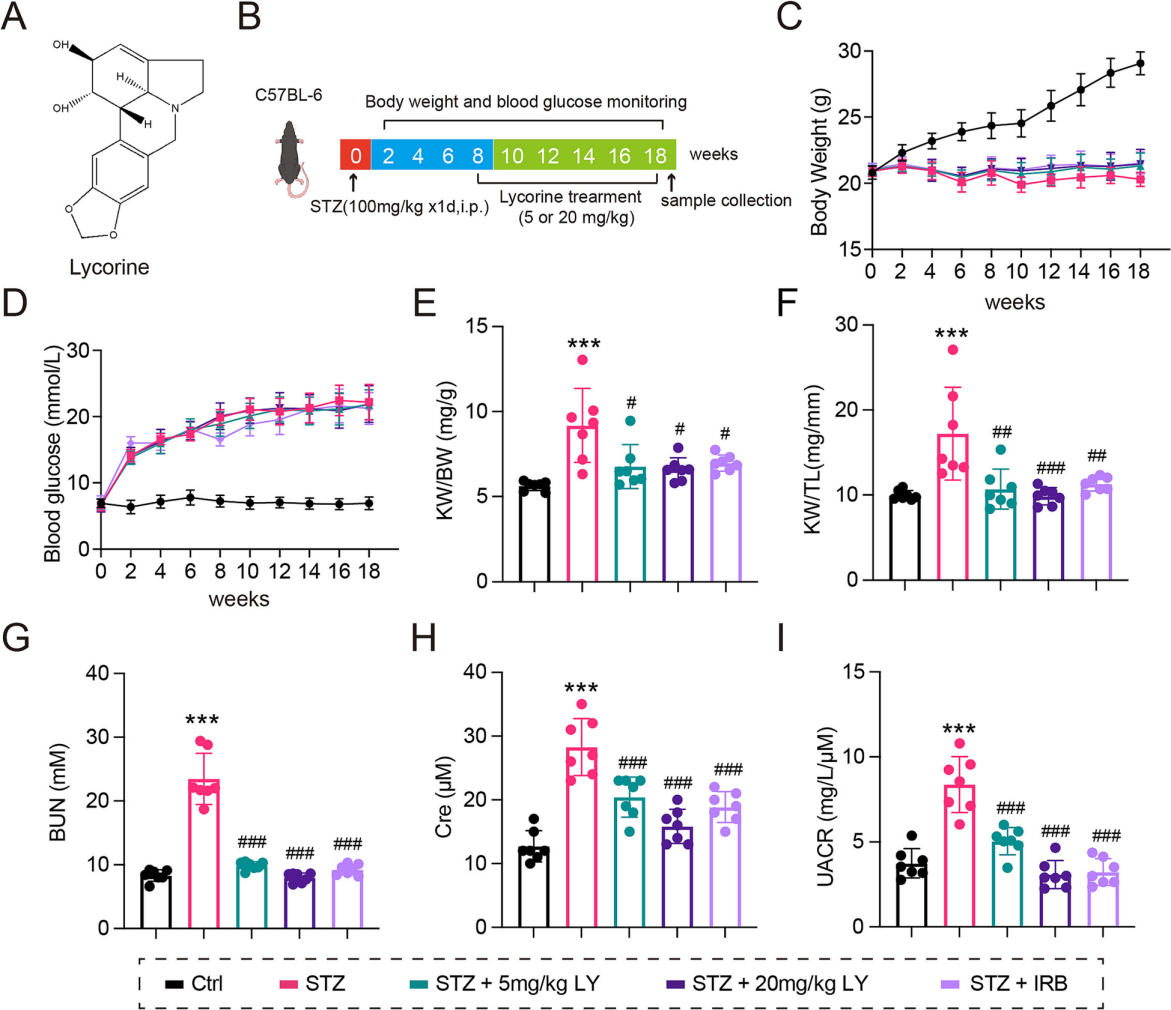
LY ameliorated diabetic nephropathy in STZ-induced type 1 diabetic mice. **A** Chemical structure of LY. **B** Schematic overview of the experimental design. **C** Body weight. **D** Blood glucose levels. **E**, **F** Kidney weight normalized to body weight (KW/BW) and to tibia length (KW/TL). **G**, **H** Serum levels of blood urea nitrogen (BUN) and creatinine (Cre). **I** Urinary albumin-to-creatinine ratio (UACR)

To assess the renoprotective potential of LY in diabetic nephropathy, we established a STZ-induced type 1 diabetic mouse model and treated the mice with LY at 5 or 20 mg/kg daily for 10 weeks (Fig. 1A-B). Irbesartan (IRB), a clinically used renoprotective agent, was included as a positive control. As expected, STZ-induced diabetic mice exhibited persistent hyperglycemia and reduced body weight gain compared with control group (Fig. 1C-D). Neither LY nor IRB significantly affected blood glucose levels or body weight changes compared with the STZ group, indicating that their renal benefits were not attributable to improved glycemic control. Nevertheless, both LY and IRB markedly alleviated diabetes-induced renal hypertrophy, as evidenced by reduced kidney weight-to-body weight and kidney weight-to-tibia length ratios (Fig. 1E-F). In addition, LY and IRB significantly attenuated the diabetes-induced increases in blood urea nitrogen, serum creatinine, and urinary albumin-to-creatinine ratio (Fig. 1G-I). These findings indicate that LY confers significantly renal protection in STZ-induced diabetic mice, and this effect occurs independently of blood glucose lowering.

### LY attenuates renal fibrosis in diabetic mice

**Fig. 2:**
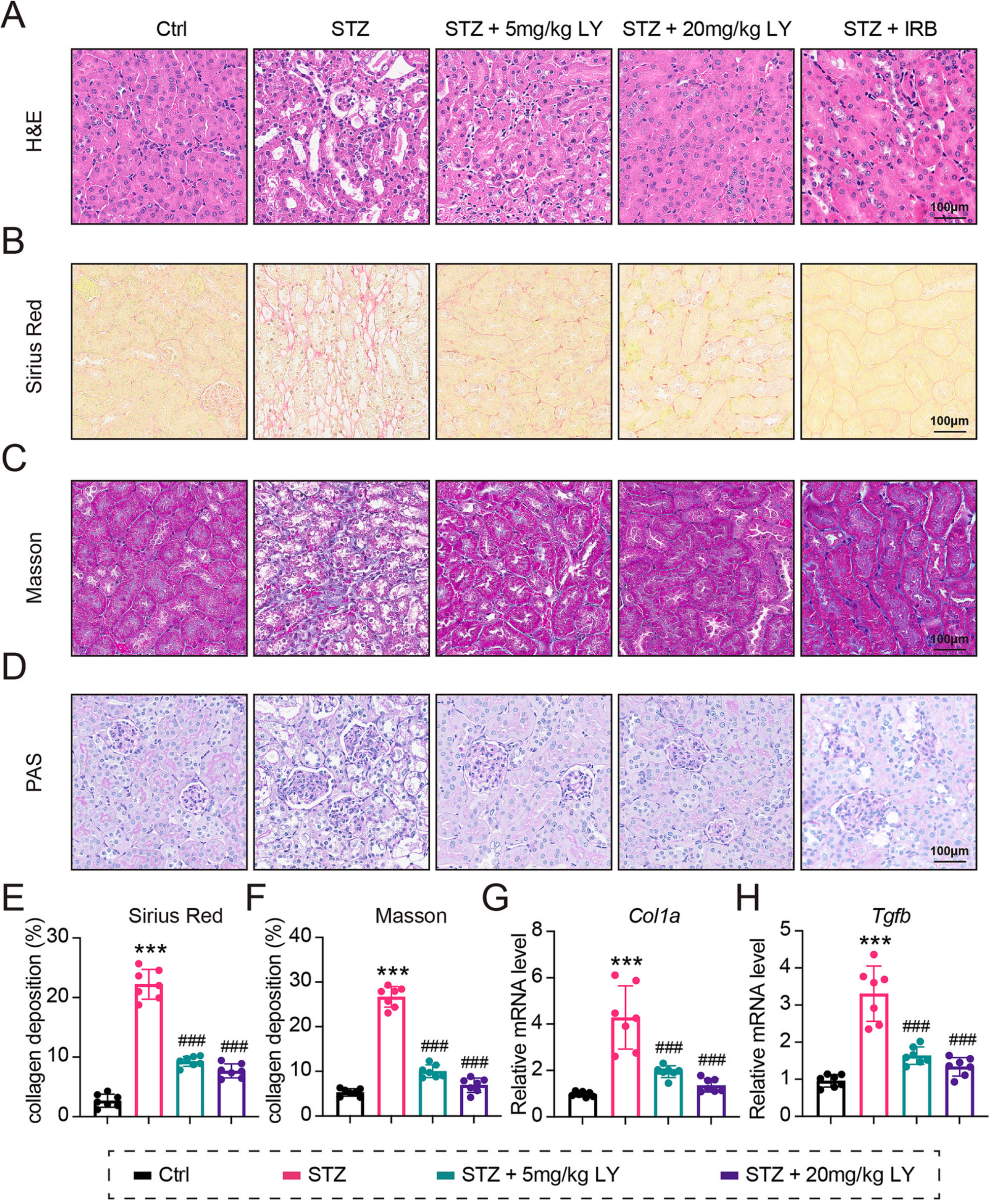
LY alleviates renal fibrosis in diabetic mice. **A** Representative hematoxylin and eosin (H&E) staining. **B** Representative Sirius Red staining. **C** Representative Masson's trichrome staining. **D** Representative Periodic acid–Schiff (PAS) staining. Scale bar = 100 μm. **E**, **F** Quantitative analysis of Sirius Red and Masson's trichrome stained areas. **G**, **H** mRNA expression levels of *Col1a1* and *TGF-β* in renal tissue, as determined by RT-qPCR

To evaluate the anti-fibrotic effect of LY in diabetic nephropathy, we performed histopathological analysis and quantified fibrosis-related gene expression in kidney tissues. Hematoxylin and eosin (H&E) staining of renal sections from STZ-induced diabetic mice revealed characteristic pathological changes, including glomerulosclerosis, mesangial cell proliferation, and matrix expansion (Fig. 2A). Sirius red and Masson's trichrome staining further demonstrated interstitial fibrosis and substantial collagen deposition in diabetic kidneys (Fig. 2B-C, Fig. 2E-F). Periodic acid-Schiff (PAS) staining also showed abundant PAS positive material accumulation in diabetic mouse kidneys, indicating glomerular basement membrane thickening and mesangial matrix expansion (Fig. 2D). Treatment with LY (5 or 20 mg/kg) significantly attenuated these diabetes-induced structural abnormalities and reduced extracellular matrix accumulation. Similar protective effects were observed in the IRB-treated group, supporting the validity of the experimental system and the anti-fibrotic efficacy of LY. Consistent with the histological findings, transcriptional analysis showed elevated mRNA levels of collagen type I alpha 1 (*col1a*) and transforming growth factor-beta (*Tgfb*) in the STZ group compared with control group. LY effectively suppressed the upregulation of these pro-fibrotic genes (Fig. 2G-H). Together, these results indicate that LY alleviates diabetes-induced renal fibrosis at both the histological and molecular levels, with effects comparable to those of the positive control IRB.

### LY reduced apoptosis, oxidative stress and inflammation in diabetic mice

**Fig. 3:**
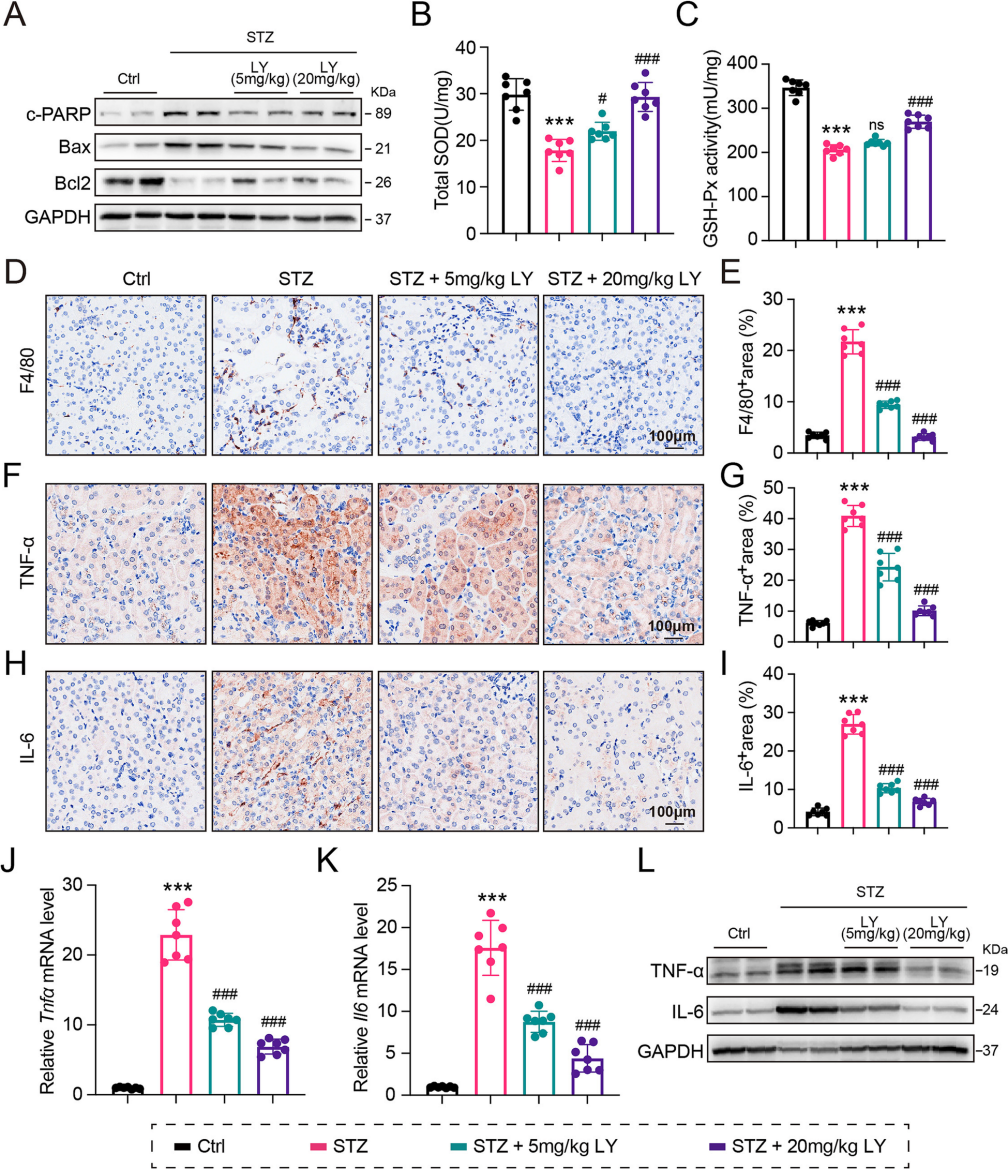
LY treatment attenuated apoptosis, oxidative stress, and inflammation in type 1 diabetic mice. **A** Protein levels of cleaved PARP, Bax, and Bcl‑2 in renal tissue assessed by Western blotting, with GAPDH serving as the loading control. **B**, **C** Serum activities of superoxide dismutase and glutathione peroxidase. **D** Representative immunohistochemical staining of F4/80‑positive macrophages in renal tissue (scale bar = 100 μm). **E** Quantification of F4/80‑positive macrophages. **F** Immunohistochemical staining of TNF‑α in renal tissue (scale bar = 100 μm). **G** Quantification of TNF‑α staining intensity. **H** Immunohistochemical staining of IL‑6 in renal tissue (scale bar = 100 μm). **I** Quantification of IL‑6 staining intensity. **J**, **K** mRNA expression levels of *Tnfa* and *Il6* in renal tissue measured by RT‑qPCR. **L** Protein expression of TNF‑α and IL‑6 in renal tissue. All Western blotting experiments were independently repeated at least three times, and representative blots are presented

To evaluate the protective effects of LY against diabetic renal injury, we systematically assessed its impact on apoptosis, oxidative stress, and inflammatory responses in kidney tissues from diabetic mice. Western blotting analysis showed that diabetic mice exhibited markedly increase expression of the pro-apoptotic proteins cleaved-PARP and Bax, together with decreased expression of the anti-apoptotic protein Bcl2, compared with control mice. Quantitative densitometric analysis further confirmed a significantly elevation of cleaved-PARP and Bax protein levels and a reduction in Bcl-2 expression in the STZ group. LY treatment significantly reversed these changes, as evidenced by decreased cleaved-PARP and Bax expression and restored Bcl-2 levels, indicating that LY attenuated diabetes-induced renal apoptosis (Fig. 3A, Fig. S1A).

To further examine oxidative stress, we measured both antioxidant enzyme activities and oxidative damage markers in renal tissues. Diabetic mice exhibited a notable reduction in total superoxide dismutase (SOD) and glutathione peroxidase (GSH-Px) activities compared with the control group. In parallel, renal reactive oxygen species (ROS) and malondialdehyde (MDA) levels were significantly elevated in diabetic mice, indicating enhanced oxidative stress and lipid peroxidation. LY administration restored SOD and GSH-Px activities and markedly reduced ROS accumulation and MDA content, suggesting that LY improved renal antioxidant capacity and suppressed diabetes-induced oxidative injury (Fig. 3B-C, Fig. S1B-C).

We further investigated the anti-inflammatory effects of LY. Immunohistochemical staining demonstrated a marked increase in F4/80-positive macrophage infiltration and elevated expression of tumor necrosis factor-alpha (TNF-α) and interleukin-6 (IL-6) in renal tissues from diabetic mice. Quantitative analysis of the staining-positive areas confirmed that F4/80, TNF-α, and IL-6 expression levels were significantly increased in the STZ group, whereas LY treatment substantially reduced macrophage infiltration and inflammatory cytokine expression (Fig. 3D-I). Consistent with these histological findings, RT-qPCR and Western blotting analyses showed that the mRNA and protein levels of TNF-α and IL-6 were markedly upregulated in diabetic kidneys (Fig. 3J-L). Quantitative densitometric analysis of Western blot bands further confirmed that LY significantly suppressed the diabetes-induced increase in TNF-α and IL-6 protein expression (Fig. S1D). Collectively, these data demonstrate that LY effectively attenuates renal apoptosis, oxidative stress, and inflammation in a mouse model of diabetic nephropathy.

### LY improves high glucose-induced cell injury in human tubular epithelial cells

To further validate the renoprotective effects of LY observed in vivo and to determine whether LY directly protects renal tubular epithelial cells under diabetic stress conditions, we established an in vitro tubular injury model using human renal tubular epithelial HK-2 cells. Cells were exposed to high-glucose plus palmitic acid (33 mM HG + 200 μM PA) with or without LY treatment for 48 h. It should be noted that the HG + PA model was not intended to fully replicate the STZ-induced type 1 diabetic mouse model. Instead, HG was used to mimic hyperglycemic stress, whereas PA was included to provide an additional metabolic/lipotoxic stimulus that exacerbates tubular epithelial injury. Thus, HG + PA stimulation was used as a robust in vitro model to better reproduce key pathological features of diabetic renal tubular injury, including apoptosis, oxidative stress, inflammatory activation, and pro-fibrotic responses.

**Fig. 4:**
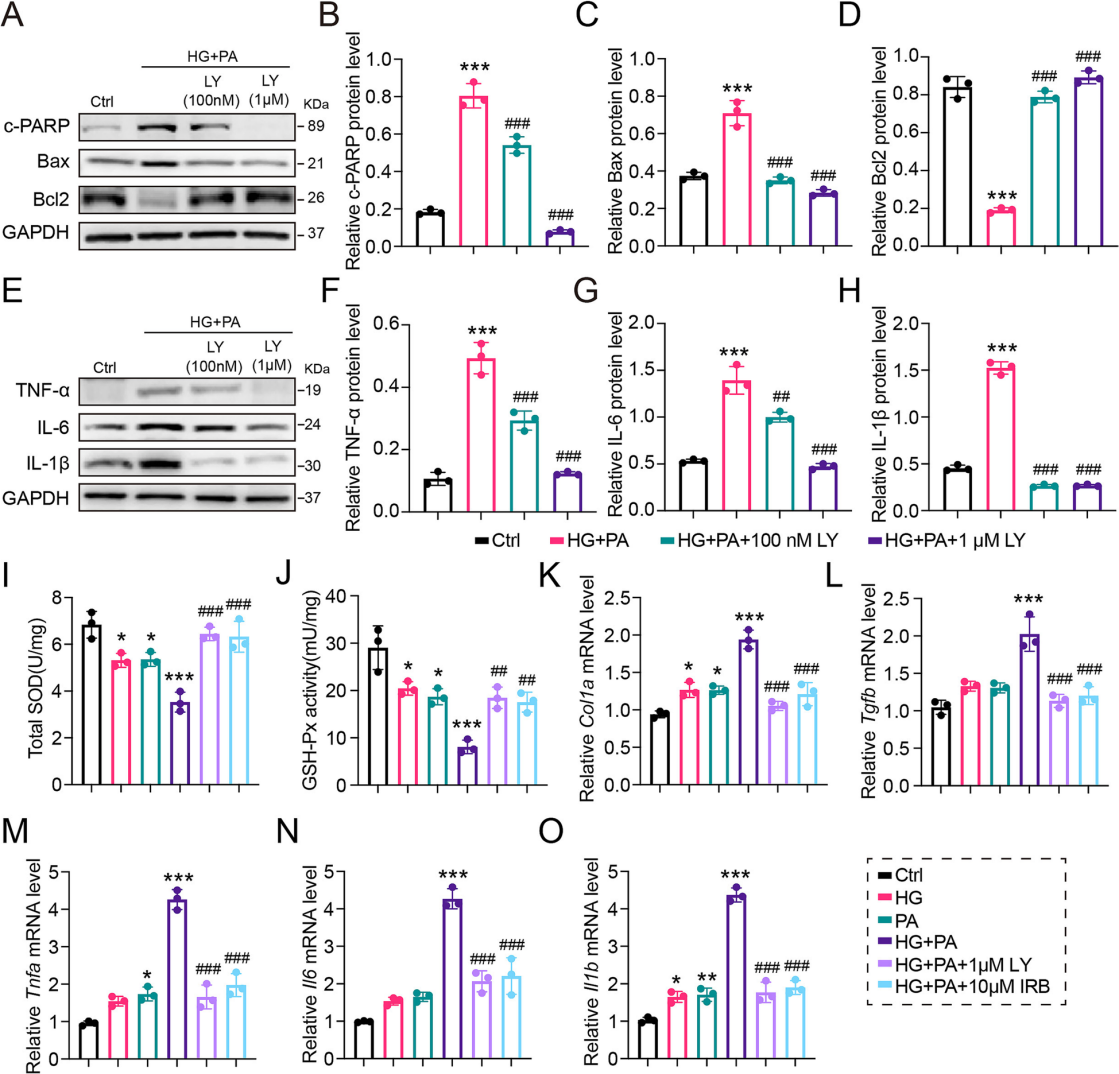
LY treatment mitigated HG + PA-induced apoptosis, oxidative stress, and inflammation in HK‑2 cells. **A** Western blotting analysis of cleaved PARP, Bax, and Bcl‑2 in HG + PA‑treated HK‑2 cells, using GAPDH as the loading control. **B**‑**D** Densitometric quantification of cleaved PARP, Bax, and Bcl‑2 protein levels. **E** Representative Western blotting showing protein expression of TNF‑α, IL‑6, and IL‑1β in HG + PA‑treated HK‑2 cells. **F**‑**H** Quantification of TNF‑α, IL‑6, and IL‑1β protein levels. **I**, **J** Cellular activities of superoxide dismutase (SOD) and glutathione peroxidase (GSH‑Px). **K**, **L** mRNA expression levels of *Col1a* and *Tgfb* measured by RT‑qPCR. **M**‑**O** Transcript levels of *Tnfa*, *Il6*, and *Il1b* analyzed by RT‑qPCR. For Western blotting, RT-qPCR, and biochemical assays, three independent biological replicates were included, and each sample was analyzed in technical triplicate. Data are expressed as mean ± SD

HG + PA stimulation significantly promoted apoptosis in HK-2 cells, as evidenced by increased protein levels of cleaved-PARP and Bax, together with decreased expression of the anti-apoptotic protein Bcl-2. Treatment with LY (0.1 and 1 μM), which showed no cytotoxicity as confirmed by the CCK-8 assay, effectively attenuated these changes and restored the balance between pro-apoptotic and anti-apoptotic regulators (Fig. 4A-D, Fig. S2A). We next assessed the effect of LY on oxidative stress. Compared with the control group, HG and PA alone induced varying degrees of cellular injury, while HG + PA caused more pronounced injury. HG + PA exposure substantially reduced total superoxide dismutase (SOD) and glutathione peroxidase activities (GSH-Px) and enhanced reactive oxygen species (ROS) and malondialdehyde (MDA) levels, whereas LY or positive control IRB co-treatment markedly restored antioxidant enzyme activities and reduced oxidative damage, indicating balanced cellular oxidative stress (Fig. 4I-J, Fig. S2B-C). Furthermore, we evaluated the expression of key pro-fibrotic and pro-inflammatory mediators. HG + PA stimulation significantly upregulated the mRNA levels of *Col1a* and *Tgfb* and this induction was substantially suppressed by LY or IRB treatment (Fig. 4K-L). At the protein level, Western blotting analysis revealed that HG + PA-induced increases in tumor necrosis factor-alpha (TNF-α), interleukin-6 (IL-6), and interleukin-1β (IL-1β) were markedly reduced by LY treatment (Fig. 4E-H). Consistently, RT-qPCR analysis confirmed that the HG + PA-induced upregulation of *Tnfα*, *Il6*, and *Il1b* mRNA was also effectively inhibited by LY or IRB (Fig. 4M-O). Collectively, these in vitro results demonstrate that LY directly protects HK-2 cells against HG + PA-induced tubular epithelial injury by suppressing apoptosis, alleviating oxidative stress, and inhibiting pro-fibrotic and pro-inflammatory responses.

### LY protected mice against diabetic nephropathy by impeding HMGB1/RAGE/NF-κB signaling axis

**Fig. 5:**
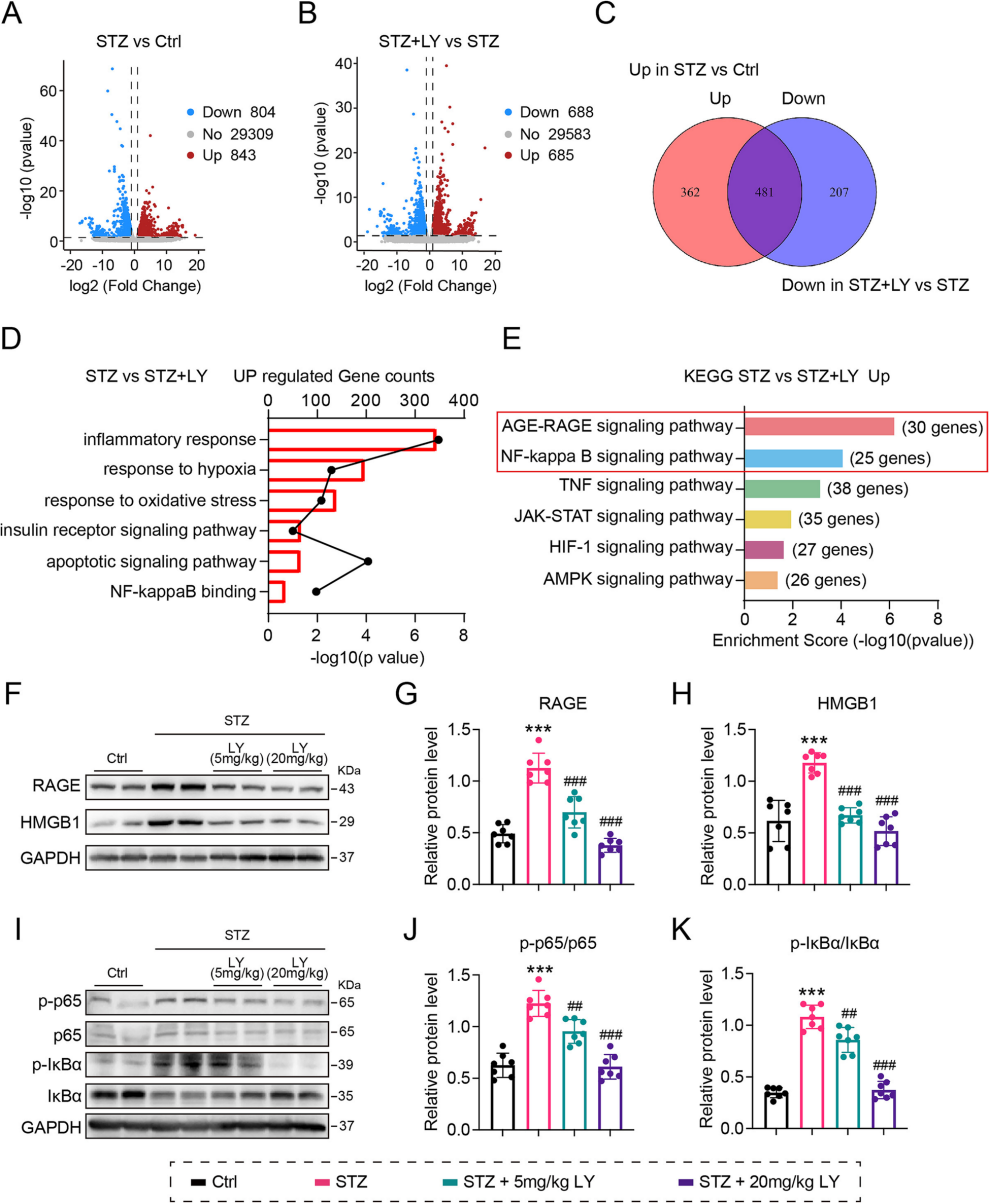
LY alleviated diabetic nephropathy by inhibiting the HMGB1/RAGE/NF-κB signaling pathway. **A**, **B** Volcano plots showing differentially expressed genes between the control and STZ groups, and between the STZ and STZ + LY groups, respectively. **C** Venn diagram illustrating the number of genes regulated by STZ alone and by STZ combined with LY. **D** Gene Ontology enrichment analysis. **E** KEGG enrichment analysis. **F**–**H** Protein levels of RAGE and HMGB1 were detected by Western blotting and quantified using ImageJ software; GAPDH was used as a loading control. **I**–**K** Protein expression levels of p‑p65, p65, p‑IκBα, and IκBα were analyzed by Western blotting and quantified with ImageJ software, with GAPDH serving as the loading control. All Western blotting experiments were independently repeated at least three times, and representative blots are presented. Quantitative densitometric analysis was performed from three independent biological replicates using ImageJ software. Data are expressed as mean ± SD

To elucidate the molecular mechanisms underlying the renoprotective effects of LY, we performed RNA-seq analysis of renal cortical tissues from control mice, STZ-induced diabetic mice, and LY-treated diabetic mice. Comparative transcriptomic profiling identified 843 upregulated and 804 downregulated genes in the STZ group compared with the controls group. In contrast, LY treatment resulted in 685 upregulated and 688 downregulated genes relative to the STZ group (Fig. 5A-B). Venn diagram analysis further identified 481 genes that were upregulated in the STZ group but downregulated following LY treatment (Fig. 5C), suggesting that LY may reverse a subset of diabetes-induced transcriptional alterations. Gene Ontology enrichment analysis showed that these 481 overlapping genes were mainly associated with biological processes related to inflammatory response, oxidative stress, and apoptosis (Fig. 5D). Consistently, KEGG pathway analysis revealed significant enrichment of the AGE-RAGE signaling pathway in diabetic complications and the NF-κB signaling pathway (Fig. 5E). To validate these transcriptomic findings at the protein level, we next examined key components of these pathways by Western blotting. Compared with the control group, STZ induction significantly increased the renal protein expression of RAGE and its ligand HMGB1, whereas LY treatment markedly suppressed these elevations (Fig. 5F-H). In parallel, immunofluorescence co-localization analysis showed enhanced co-localization of RAGE and HMGB1 in renal tissues from STZ-induced diabetic mice, indicating increased activation of the HMGB1/RAGE signaling axis under diabetic conditions. Notably, LY treatment markedly reduced the fluorescence intensity and co-localization of RAGE and HMGB1, further supporting its inhibitory effect on this pathway (Fig. S3). In line with activation of the NF-κB pathway, the phosphorylation levels of p65 and IκBα were also markedly increased in diabetic mice; however, these phosphorylation events were substantially reduced after LY administration (Fig. 5I-K). Together, these findings indicate that LY exerts protective effects against diabetic nephropathy, at least in part, by suppressing the HMGB1/RAGE/NF-κB signaling axis.

### LY attenuates diabetic nephropathy by inhibiting RAGE

**Fig. 6:**
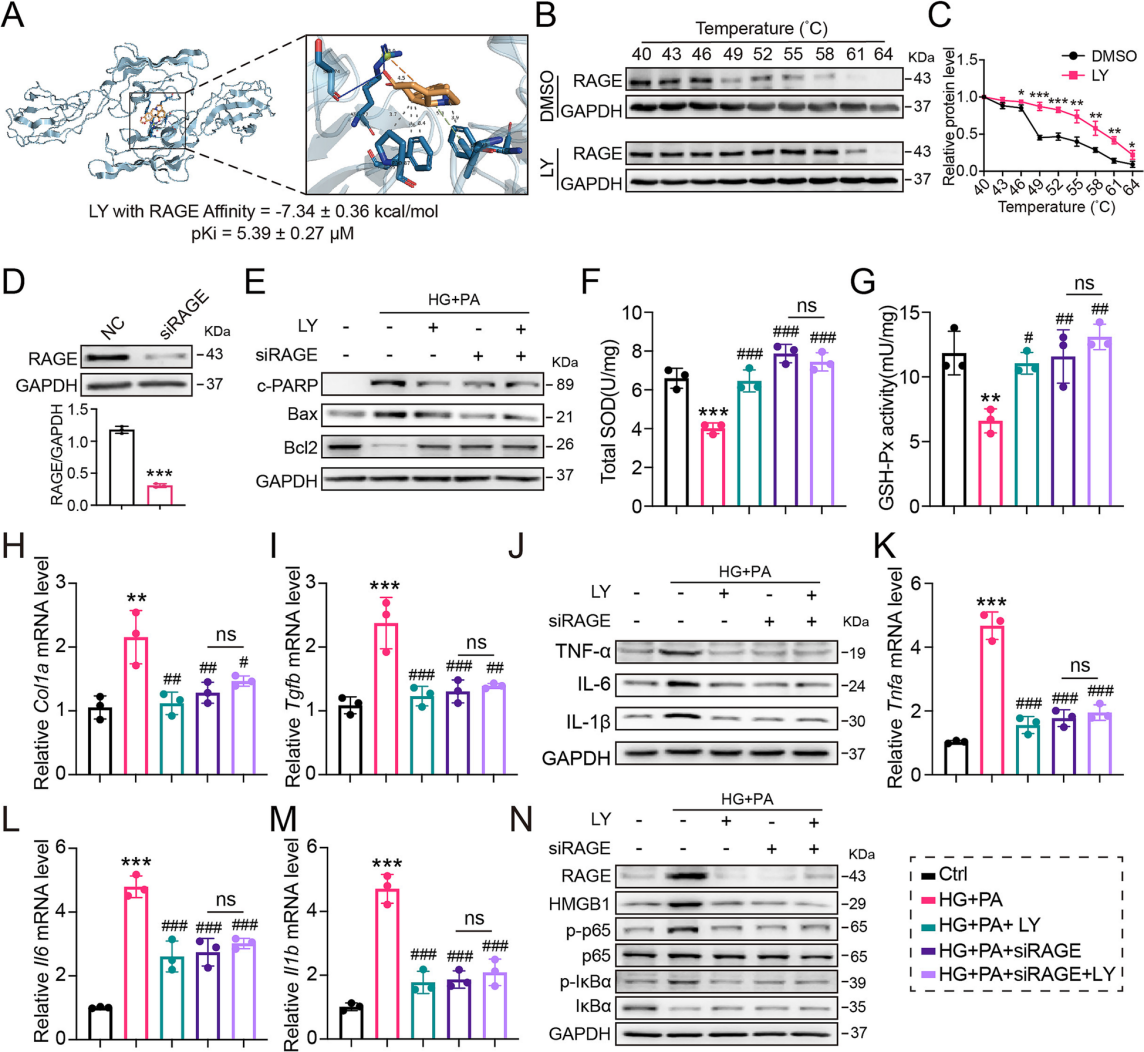
LY ameliorates diabetic nephropathy through RAGE inhibition. **A** Molecular docking model of LY bound to RAGE. **B** CETSA analysis of RAGE thermal stability in the presence or absence of LY. **C** Quantification of RAGE protein expression levels. **D** Validation of RAGE knockdown efficiency via Western blotting. Representative bands and quantitative analysis of RAGE protein expression are shown. **E** Western blotting analysis of cleaved PARP, Bax, and Bcl‑2, normalized to GAPDH. **F**-**G** Total SOD activity and GSH‑Px levels. **H**–**I** Relative mRNA expression of *Col1a1* and *Tgfb* measured by RT‑qPCR and normalized to *Gapdh*. **J** Protein levels of TNF‑α, IL‑6, and IL‑1β assessed by Western blotting with GAPDH as loading control. **K**–**M** Relative mRNA levels of *Tnfα*, *Il6*, and *Il1b* determined by RT‑qPCR and normalized to *Gapdh*. **N** Western blotting analysis of RAGE, HMGB1, p‑p65, p65, p‑IκBα, and IκBα protein expression; GAPDH served as loading control. All Western blotting experiments were independently repeated at least three times, and representative blots are presented. Quantitative densitometric analysis was performed from three independent biological replicates using ImageJ software. For RT-qPCR and biochemical assays, three independent biological replicates were included, and each sample was analyzed in technical triplicate. Data are expressed as mean ± SD

To further investigate the molecular target through which LY exerts its renoprotective effects, we focused on the receptor for advanced glycation end products (RAGE), a key mediator in the pathogenesis of diabetic nephropathy. Molecular docking analysis revealed that LY could bind to RAGE with a binding free energy of −7.34 ± 0.36 kcal/mol, corresponding to a predicted inhibition constant (pKi) of 5.39 ± 0.27 μM, suggesting a favorable binding affinity between LY and RAGE (Fig. 6A). To further validate this interaction, surface plasmon resonance (SPR) analysis was performed. The SPR results showed that LY directly bound to recombinant RAGE protein in a concentration-dependent manner, with an equilibrium dissociation constant (K_D_) of 12.5 μM, further confirming the direct interaction between LY and RAGE (Fig. S4A). Consistently, the cellular thermal shift assay demonstrated that LY treatment significantly enhanced the thermal stability of RAGE, as evidenced by increased RAGE protein retention at elevated temperatures. Quantitative analysis of RAGE protein levels further confirmed that LY markedly stabilized RAGE against thermal denaturation (Fig. 6B-C).

To elucidate the functional significance of RAGE inhibition in LY-mediated renoprotection, we next performed RAGE knockdown experiments in vitro. Western blotting and quantitative analysis confirmed that siRNA-mediated RAGE silencing markedly reduced RAGE protein expression compared with the negative control group, validating the efficiency of RAGE knockdown (Fig. 6D). Functionally, RAGE silencing effectively attenuated HG + PA-induced apoptosis, as indicated by decreased protein expression of cleaved-PARP and Bax, together with restoration of the anti-apoptotic protein Bcl-2. Notably, LY treatment did not produce additional anti-apoptotic effects in RAGE-deficient cells, suggesting that the anti-apoptotic activity of LY is largely dependent on the presence of RAGE (Fig. 6E, Fig. S4B). Similarly, RAGE knockdown alone improved cellular antioxidant capacity, as reflected by increased total superoxide dismutase and glutathione peroxidase activities, reaching levels comparable to those observed in LY-treated RAGE-expressing cells (Fig. 6F-G). In parallel, the mRNA expression levels of pro-fibrotic markers *Col1a1* and *Tgfb* were markedly reduced following *Rage* silencing (Fig. 6H-I). However, LY administration failed to further enhanced antioxidant enzyme activities or further suppress pro-fibrotic gene expression in RAGE-deficient cells, indicating that RAGE is required for these protective effects of LY. Furthermore, RAGE ablation substantially suppressed the production of pro-inflammatory cytokines, including TNF-α, IL-6, and IL-1β, under HG + PA stimulation (Fig. 6J-M, Fig. S4C). Consistently, Western blotting analysis showed that RAGE knockdown markedly diminished the protein levels of HMGB1, p-p65, and p-IκBα. Quantitative analysis further demonstrated significant reductions in HMGB1 expression, the p-p65/p65 ratio, and the p-IκBα/IκBα ratio after RAGE silencing. Importantly, LY treatment did not further suppress HMGB1 expression or NF-κB pathway activation in RAGE-silenced cells (Fig. 6N, Fig. S4D). Collectively, these findings demonstrate that RAGE serves as a direct molecular target of LY. LY directly binds to RAGE and stabilizes its protein structure, while the loss of RAGE largely abolishes the additional protective effects of LY against HG + PA-induced apoptosis, oxidative stress, fibrosis, and inflammation. These results indicate that the renoprotective effects of LY are largely mediated through inhibition of the HMGB1/RAGE/NF-κB signaling axis.

## Discussion

**Fig. 7:**
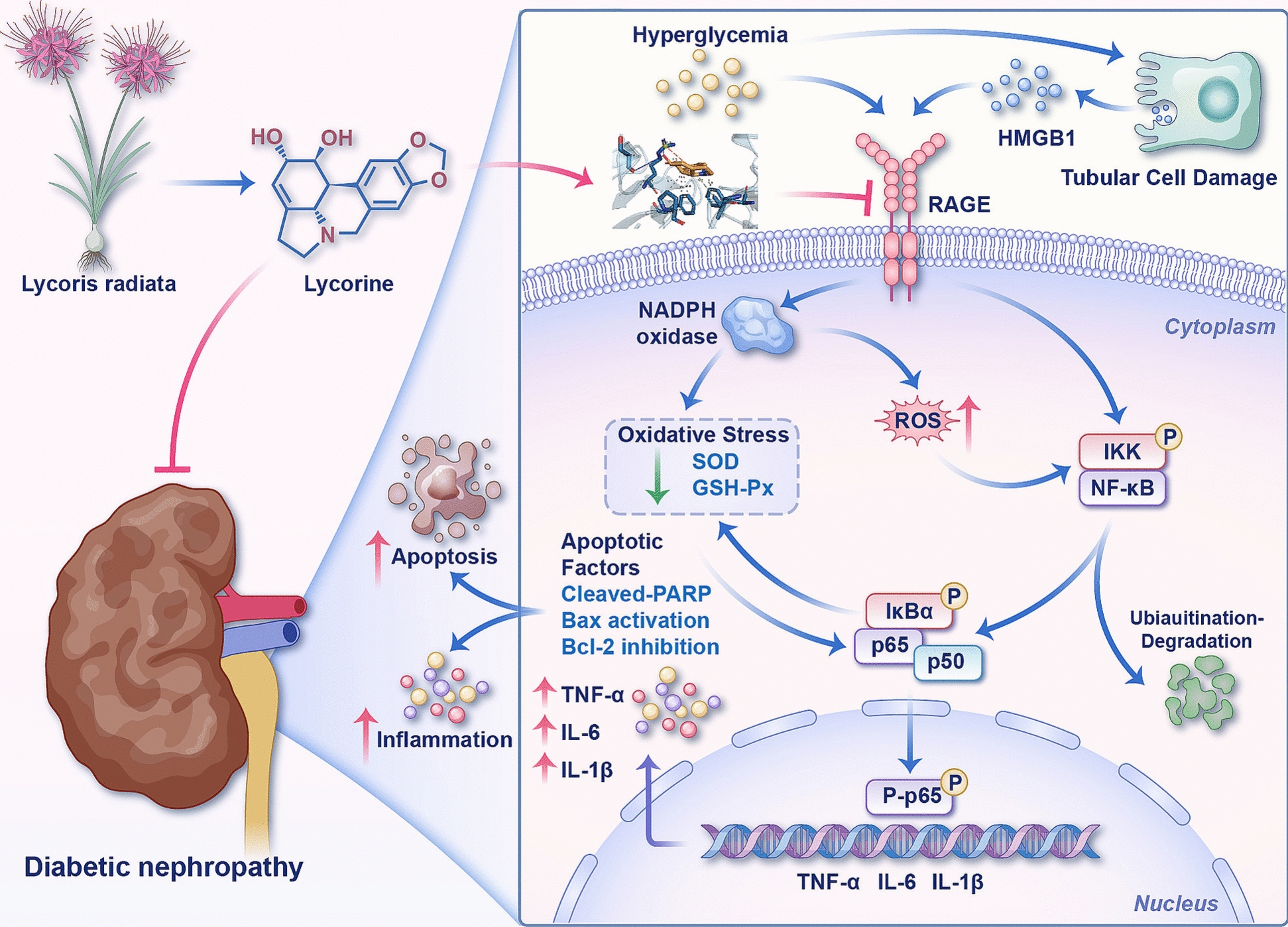
Schematic illustration of the protective effects of LY against diabetic nephropathy. Lycorine (LY), derived from *Lycoris radiata*, directly binds to RAGE and inhibits hyperglycemia-induced activation of the RAGE signaling pathway in renal tubular cells. By suppressing RAGE activation, LY attenuates downstream ROS generation and oxidative stress, restores antioxidant defenses including SOD and GSH-Px, and inhibits activation of the IKK/IκBα/NF-κB pathway. Consequently, LY reduces NF-κB p65 phosphorylation and nuclear transcriptional activity, leading to decreased expression of pro-inflammatory cytokines, including TNF-α, IL-6, and IL-1β. In addition, LY alleviates apoptosis by reducing cleaved-PARP and Bax activation while restoring Bcl-2 expression. Together, LY protects against diabetic nephropathy by targeting the HMGB1/RAGE/NF-κB axis and suppressing oxidative stress, inflammation, and apoptosis. Figure created with www.home-for-researchers.com

Diabetic nephropathy remains a major global health challenge because its progression is driven by complex and interconnected metabolic, inflammatory, oxidative, apoptotic, and fibrotic pathways [8, 24]. Although numerous natural products have shown protective effects in preclinical models of diabetic kidney disease, their clinical translation has often been limited by insufficient identification of precise molecular targets, incomplete mechanistic characterization, and inadequate evaluation of translational feasibility [11, 12, 17]. In this study, we show that LY, a natural isoquinoline alkaloid derived from the *Amaryllidaceae* family, confers broad renoprotective effects in streptozotocin (STZ)-induced diabetic mice and in HG + PA-stimulated HK-2 cells. LY alleviated renal dysfunction, pathological injury, fibrosis, apoptosis, oxidative stress, and inflammation. Mechanistically, our data identify RAGE as a direct molecular target of LY and demonstrate that LY suppresses the HMGB1/RAGE/NF-κB signaling axis, thereby attenuating inflammatory and injury-related responses in diabetic nephropathy (Fig. 7).

An important of this study is that LY improved renal injury without significantly lowering blood glucose levels. In STZ-induced diabetic mice, persistent hyperglycemia and diabetes-associated body weight changes were observed, and LY treatment did not significantly alter blood glucose levels or body weight compared with STZ group. Nevertheless, LY markedly reduced renal hypertrophy, blood urea nitrogen, serum creatinine, urinary albumin-to-creatinine ratio, renal fibrosis, apoptosis, oxidative stress, macrophage infiltration, and inflammatory cytokine expression. These findings indicate that the renoprotective effect of LY is largely independent of systemic glycemic control. Therefore, LY should not be interpreted as a glucose-lowering antidiabetic agent in the present model. Instead, its protective effect appears to be medicated mainly through direct inhibition of kidney injury-related inflammatory signaling. This distinction is important because renal inflammation, oxidative stress, and fibrosis may continue to drive diabetic nephropathy progression even when hyperglycemia is partially controlled. Thus, LY may have potential as an adjunctive renoprotective therapy rather than as a stand-alone antidiabetic treatment.

The relationship between the transcriptomics-identified AGE-RAGE pathway and the experimentally validated HMGB1/RAGE/NF-κB signaling axis also warrants clarification. RAGE is a muti-ligand pattern-recognition receptor that serves as a central molecular hub linking diabetic metabolic stress to inflammatory tissue injury [14]. AGEs are classical RAGE ligands generated under chronic hyperglycemic conditions and are widely recognized as major upstream triggers of RAGE activation in diabetic complications. Therefore, the enrichment of the AGE-RAGE signaling pathway in our RNA-seq analysis reflects the activation of RAGE-centered pathogenic signaling under diabetic conditions. However, RAGE activation in diabetic nephropathy is not limited to AGEs. HMGB1, a damage-associated molecular pattern released during cellular stress, inflammation, and tissue injury, is another important RAGE ligand. Compared with AGEs, which mainly represent hyperglycemia-driven metabolic stress, HMGB1 more strongly reflects injury-associated sterile inflammation and inflammatory amplification [9, 18]. Thus, AGEs and HMGB1 are not mutually exclusive RAGE ligands. Rather, they may act sequentially, cooperatively, or synergistically during diabetic nephropathy progression. AGEs may initiate or sustain RAGE activation under chronic hyperglycemic stress, while HMGB1 released from injured renal cells or infiltrating inflammatory cells may further amplify RAGE-dependent NF-κB activation. Both ligands converge on RAGE and promote downstream inflammatory signaling, oxidative stress, apoptosis, and fibrosis.

Based on this rationale, our transition from transcriptomic AGE-RAGE pathway enrichment to focused validation of the HMGB1/RAGE/NF-κB axis is mechanistically justified. RNA-seq analysis identified AGE-RAGE and NF-κB pathway as key pathways regulated by LY in diabetic kidneys. These omics findings suggested that RAGE-dependent inflammatory signaling is a central pathway affected by LY. We therefore further examined HMGB1/RAGE/NF-κB signaling, an inflammation-amplifying branch of RAGE activation that is highly relevant to sterile renal inflammation. Western blotting showed that STZ-induced diabetes increased renal RAGE and HMGB1 expression and activated NF-κB signaling, as indicated by increased phosphorylation of p65 and IκBα. LY treatment significantly suppressed these changes. These results support the concept that LY alleviates diabetic renal injury by targeting RAGE and interrupting HMGB1/RAGE-mediated NF-κB activation.

Our findings are consistent with previous studies showing that RAGE-dependent signaling plays a critical role in diabetic kidney injury. RAGE activation has been implicated in renal inflammation, oxidative stress, fibrosis, and progressive functional decline in diabetic nephropathy [18, 22, 28]. In particular, activation of RAGE by AGEs or HMGB1 can promote NF-κB signaling and induce the transcription of pro-inflammatory cytokines, including TNF-α, IL-6, and IL-1β. In agreement with this mechanism, we observed that LY reduced renal and cellular inflammatory responses, decreased oxidative stress, attenuated apoptosis, and inhibited pro-fibrotic gene expression. Importantly, our study further extends previous work by identifying LY as a direct RAGE-targeting compound. Molecular docking predicted a favorable binding between LY and the RAGE, and CETSA confirmed LY-induced thermal stabilization of RAGE in cells. Moreover, RAGE knockdown largely abolished the additional protective effects of LY on apoptosis, antioxidant enzyme activity, fibrotic gene expression, inflammatory cytokine production, and NF-κB activation. These results indicate that RAGE is not only a pathway marker affected by LY but also a functionally indispensable target required for LY-mediated renal protection.

The present findings also expand current knowledge regarding the pharmacological actions of LY. Previous studies have reported that LY possesses anti-inflammatory, anti-fibrotic, antiviral, anticancer, and organ-protective properties. For example, LY has been shown to ameliorate bleomycin-induced pulmonary fibrosis by inhibiting NLRP3 inflammasome activation and pyroptosis, and to protect against inflammatory cardiac injury through modulation of inflammation-related signaling pathways [10, 19]. However, the therapeutic role of LY in diabetic nephropathy and its direct molecular target in diabetic kidney injury have remained insufficiently defined. Our study provides new evidence that LY protects against diabetic nephropathy by directly targeting RAGE and suppressing downstream HMGB1/RAGE/NF-κB signaling. Therefore, the novelty of our work lies not only in demonstrating the renoprotective activity of LY, but also in establishing a target-based mechanism that links LY to a defined pathogenic receptor in diabetic nephropathy.

Several natural products have also been reported to ameliorate diabetic nephropathy by regulating RAGE/NF-κB-related pathways or other inflammation-associated mechanisms. For instance, geniposide was reported to improve diabetic nephropathy in type 2 diabetic mice by targeting the AGEs-RAGE-dependent inflammatory pathway. Other natural compounds, including wogonin, glabridin, and isoquercitrin, have been shown to protect against diabetic kidney injury by regulating autophagy, apoptosis, ferroptosis, oxidative stress, inflammation, and fibrotic signaling [12, 17, 24]. These studies collectively support the concept that natural products represent a valuable source of candidate molecules for intervening in inflammation-driven diabetic kidney injury. Compared with many previous studies that mainly demonstrated pathway-level inhibition, our work provides additional target-level evidence by showing that LY directly interacts with RAGE and that RAGE knockdown eliminates the additive protective effects of LY. This target-dependence strengthens the mechanistic significance of LY and distinguishes it from natural compounds whose direct molecular targets remain unclear.

The fact that LY did not lower blood glucose has important implications for clinical translation. On one hand, this may be viewed as a limitation because persistent hyperglycemia remains the primary upstream driver of diabetic nephropathy, and LY alone would not be expected to replace standard glucose-lowering therapy. Therefore, LY may not be suitable as monotherapy for diabetes or diabetic nephropathy in patients requiring glycemic control. On the other hand, the glucose-independent protective effect of LY may represent a clinically relevant advantage. Many patients with diabetic nephropathy continue to experience renal inflammation, oxidative stress, albuminuria, and fibrosis despite improved glycemic control. Thus, a therapy that directly targets kidney injury pathways may provide additional benefit when combined with standard treatments. In this context, LY may be developed as an adjunctive renoprotective agent used in combination with glucose-lowering drugs, SGLT2 inhibitors, RAAS blockade, mineralocorticoid receptor antagonists, or other clinically established therapies for diabetic kidney disease. Future studies should evaluate whether LY can provide additive or synergistic protection when combined with current standard-of-care treatments.

From a translational perspective, LY has several attractive features, including its natural origin, small-molecule structure, anti-inflammatory activity, and direct target engagement with RAGE. Nevertheless, substantial work is still required before LY can be advanced toward clinical application. First, the pharmacokinetic and pharmacodynamic properties of LY need to be systematically characterized, including oral absorption, plasma half-life, metabolic stability, renal tissue distribution, renal accumulation, and in vivo target engagement. Second, because diabetic nephropathy requires long-term treatment, chronic safety evaluation is essential. Potential hepatotoxicity, nephrotoxicity, hematological toxicity, and off-target effects should be carefully assessed. Third, the druggability of LY requires further optimization. Although LY is a bioactive natural alkaloid, its solubility, bioavailability, metabolic stability, and therapeutic window may limit direct clinical translation. Structural modification, prodrug design, sustained-release formulations, or kidney-targeted delivery systems may help improve renal exposure, enhance efficacy, and reduce systemic toxicity.

Several limitations should be acknowledged. First, although the STZ-induced diabetic mouse model is widely used to study diabetic nephropathy, it mainly reflects type 1 diabetes driven by insulin deficiency and hyperglycemia. The therapeutic effect of LY should be further validated in type 2 diabetic nephropathy models, such as db/db mice or high-fat diet/STZ-induced diabetic models. Second, the in vitro HG + PA model represents combined hyperglycemic and lipotoxic stress in renal tubular epithelial cells rather than a complete cellular equivalent of STZ-induced type 1 diabetes. Therefore, additional studies using HG-alone stimulation, primary renal tubular epithelial cells, podocytes, mesangial cells, and human kidney organoids are needed to further confirm the generalizability of LY-mediated RAGE inhibition. Third, although our data support RAGE as a direct and indispensable target of LY, the precise binding site and whether LY interferes with RAGE ligand binding, receptor dimerization, internalization, or downstream signalosome assembly remain to be determined. Fourth, the relative contribution of different RAGE ligands, particularly AGEs and HMGB1, should be further clarified using ligand-specific intervention strategies.

## Conclusion

In summary, this study demonstrates that LY alleviates diabetic nephropathy without lowering blood glucose, indicating a glucose-independent renoprotective mechanism. Mechanistically, LY directly targets RAGE and suppresses HMGB1/RAGE-mediated NF-κB activation, thereby reducing renal inflammation, oxidative stress, apoptosis, and fibrosis. By clarifying the relationship between AGE-RAGE pathway enrichment and HMGB1/RAGE/NF-κB signaling, our findings provide a coherent mechanistic framework in which RAGE acts as a central convergence point for metabolic stress and inflammatory amplification in diabetic nephropathy. These results support LY as a promising RAGE-targeting natural compound for diabetic kidney disease, particularly as a potential adjunctive therapy combined with current glucose-lowering and renoprotective treatments. However, further studies are required to define its pharmacokinetics, long-term safety, druggability, and efficacy in additional diabetic models and human-relevant experimental systems.

## Supplementary Information

Supplementary Material 1: Fig. S1. LY attenuates renal apoptosis, oxidative stress, and inflammation in STZ-induced diabetic mice.Quantitative analysis of apoptosis-related proteins, including cleaved PARP, Bax, and Bcl2, in renal tissues from control mice, STZ-induced diabetic mice, and diabetic mice treated with LY at 5 or 20 mg/kg.Quantification of reactive oxygen specieslevels in renal tissues.Measurement of malondialdehydelevels in renal tissues.Quantitative analysis of inflammatory cytokine protein levels, including TNF-α and IL-6. Data are presented as mean ± SD

Supplementary Material 2: Fig. S2. LY protects against HG + PA-induced cytotoxicity, ROS accumulation, and lipid peroxidation in renal tubular epithelial cells.Cell viability was assessed by CCK-8 assay in cells treated with LYin the presence or absence of HG + PA stimulation.Representative fluorescence images showing intracellular ROS production detected by DCFH-DA staining. Scale bar = 50 μm.Quantification of MDA levels in different treatment groups. Data are presented as mean ± SD. Each dot represents an independent biological replicate

Supplementary Material 3: Fig. S3. LY prevebts the colocalization of RAGE and HMGB1 in STZ-induced mice.Representative double immunofluorescence staining images showing the colocalization of RAGEand HMGB1in renal tissues. DAPIwas used for nuclear staining.Fluorescence-intensity quantification of RAGE, HMGB1, and DAPIin renal tissues from T1DM mice

Supplementary Material 4: Fig. S4. LY directly binds to RAGE and RAGE knockdown abolishes the additional protective effects of LY in HG + PA-treated cells.Surface plasmon resonanceanalysis showing the concentration-dependent binding of LY to recombinant RAGE protein. K_D_ = 1.25 × 10⁻⁵ M.Quantitative analysis of apoptosis-related proteins, including cleaved PARP, Bax, and Bcl2, in control cells, HG + PA-treated cells, and cells subjected to LY treatment and/or RAGE knockdown.Quantitative analysis of inflammatory cytokine protein levels, including TNF-α, IL-6, and IL-1β.Quantitative analysis of HMGB1/RAGE/NF-κB signaling-related proteins, including RAGE, HMGB1, p-p65/p65, and p-IκBα/IκBα. Data are presented as mean ± SD.

## Data Availability

The datasets used and/or analyzed during the current study are available from the corresponding author on reasonable request.

## Abbreviations

BUN: Blood urea nitrogen
CETSA: Cellular thermal shift assay
Cre: Urinary creatinine
DN: Diabetic nephropathy
H&E: Hematoxylin–eosin
HG: High glucose
HK-2: Human renal tubular epithelial
HMGB1: High mobility group box 1
LY: *Lycorine*
PA: Palmitic acid
PAS: Periodic acid-Schiff
RAGE: Receptor for advanced glycation end products
SPR: Surface plasmon resonance
STZ: Streptozotocin
T1MD: Type 1 diabetes mellitus
UACR: Urinary albumin-to-creatinine ratio
