# Supplementary figures and images for: Lycorine ameliorates diabetic nephropathy by targeting RAGE and inhibiting the HMGB1/RAGE/NF-κB signaling axis
Source: Chin Med. 2026 Jul 16;21:193. doi: 10.1186/s13020-026-01469-y (PMC13374191; doi:10.1186/s13020-026-01469-y)

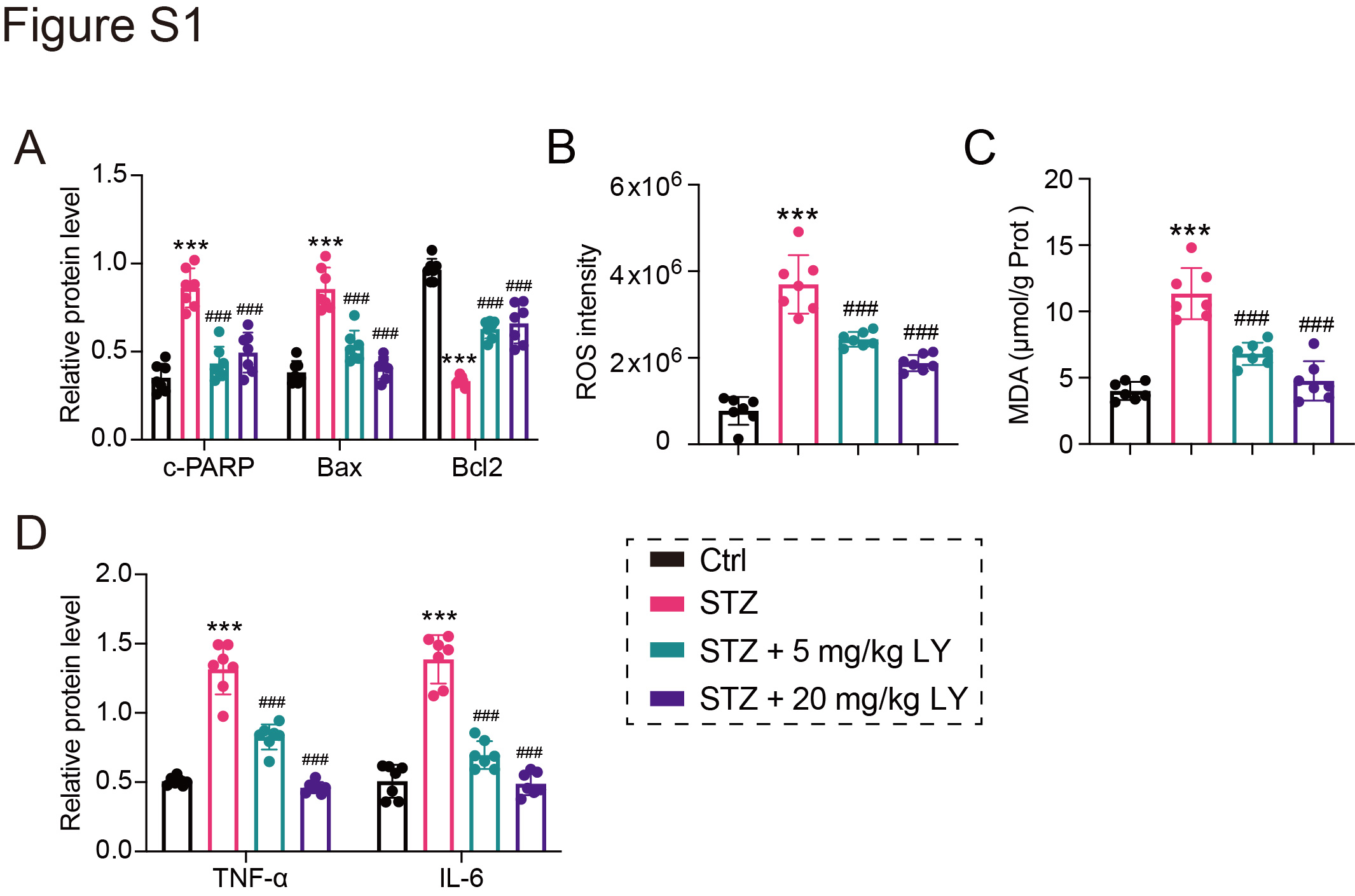

Supplement: Supplementary file 1 — Supplementary Material 1: Fig. S1. LY attenuates renal apoptosis, oxidative stress, and inflammation in STZ-induced diabetic mice.Quantitative analysis of apoptosis-related proteins, including cleaved PARP, Bax, and Bcl2, in renal tissues from control mice, STZ-induced diabetic mice, and diabetic mice treated with LY at 5 or 20 mg/kg.Quantification of reactive oxygen specieslevels in renal tissues.Measurement of malondialdehydelevels in renal tissues.Quantitative analysis of inflammatory cytokine protein levels, including TNF-α and IL-6. Data are presented as mean ± SD [file 13020_2026_1469_MOESM1_ESM.jpg]

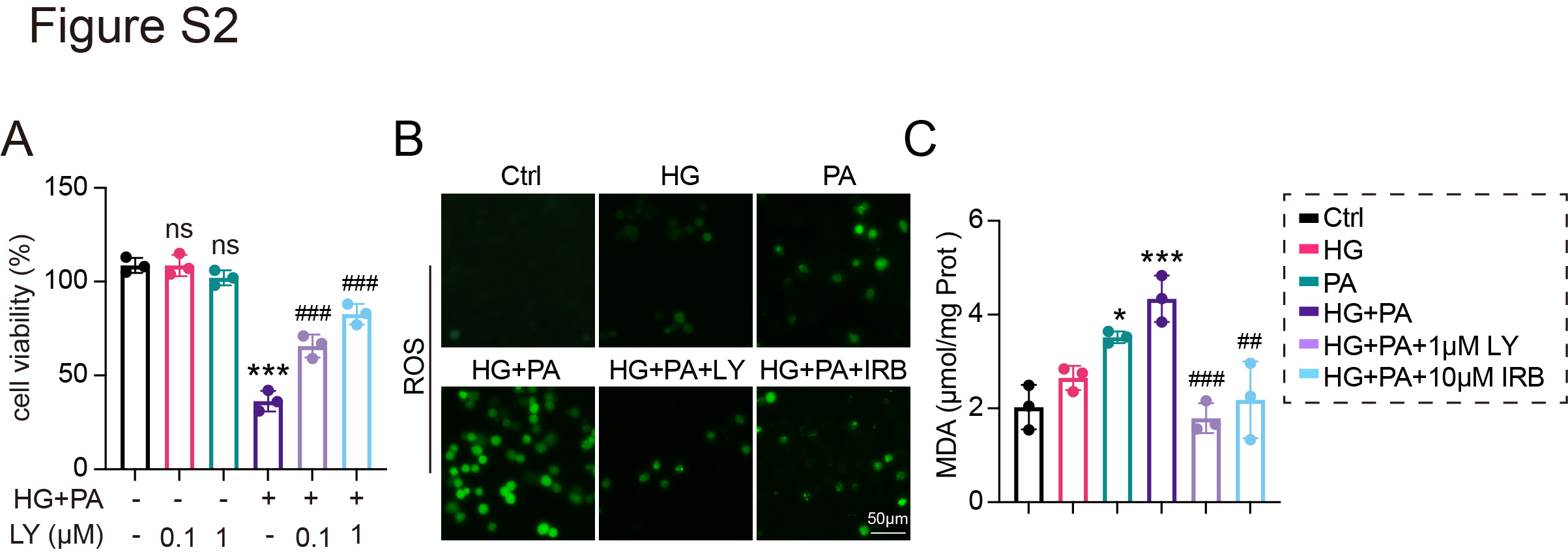

Supplement: Supplementary file 2 — Supplementary Material 2: Fig. S2. LY protects against HG + PA-induced cytotoxicity, ROS accumulation, and lipid peroxidation in renal tubular epithelial cells.Cell viability was assessed by CCK-8 assay in cells treated with LYin the presence or absence of HG + PA stimulation.Representative fluorescence images showing intracellular ROS production detected by DCFH-DA staining. Scale bar = 50 μm.Quantification of MDA levels in different treatment groups. Data are presented as mean ± SD. Each dot represents an independent biological replicate [file 13020_2026_1469_MOESM2_ESM.jpg]

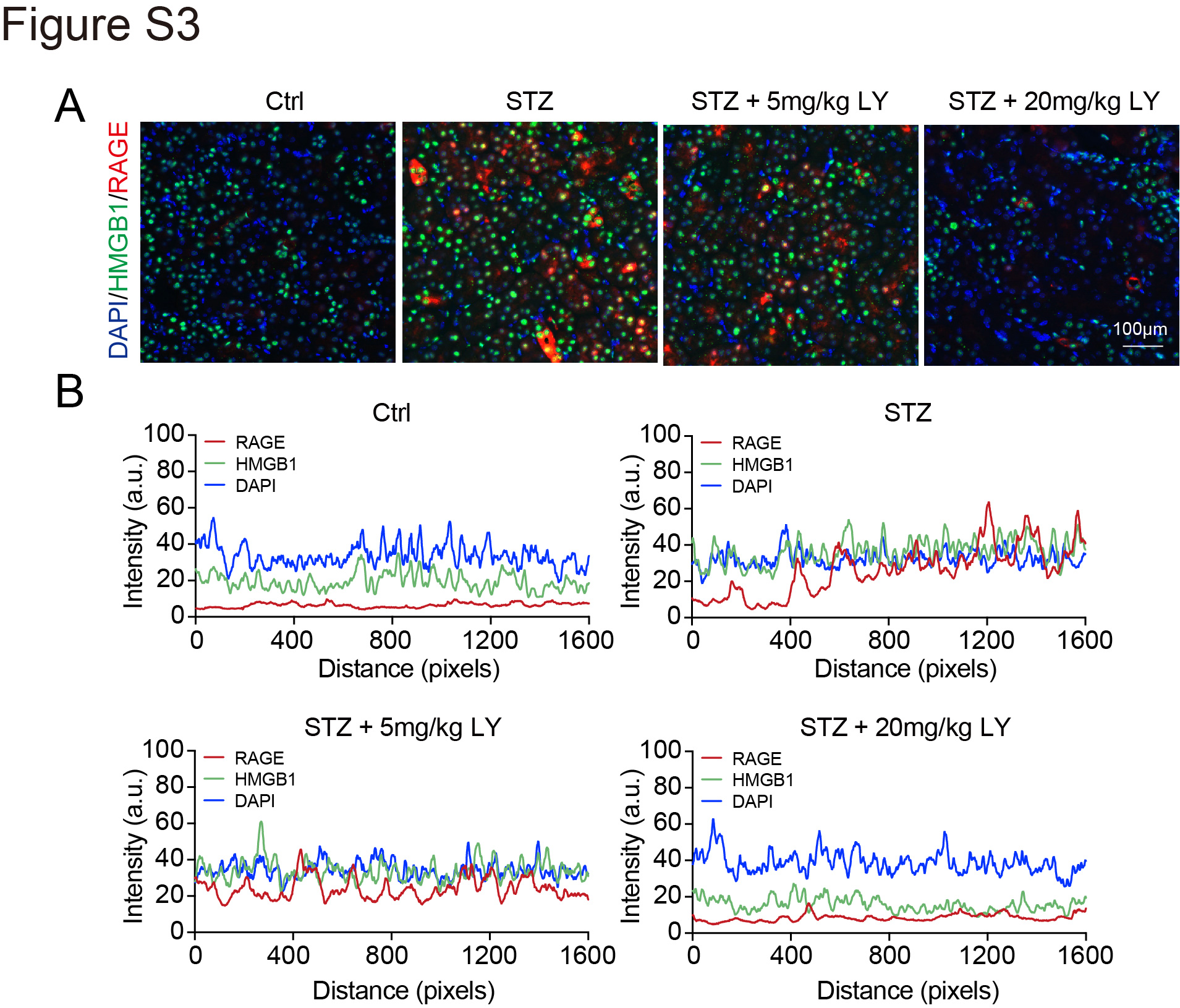

Supplement: Supplementary file 3 — Supplementary Material 3: Fig. S3. LY prevebts the colocalization of RAGE and HMGB1 in STZ-induced mice.Representative double immunofluorescence staining images showing the colocalization of RAGEand HMGB1in renal tissues. DAPIwas used for nuclear staining.Fluorescence-intensity quantification of RAGE, HMGB1, and DAPIin renal tissues from T1DM mice [file 13020_2026_1469_MOESM3_ESM.jpg]

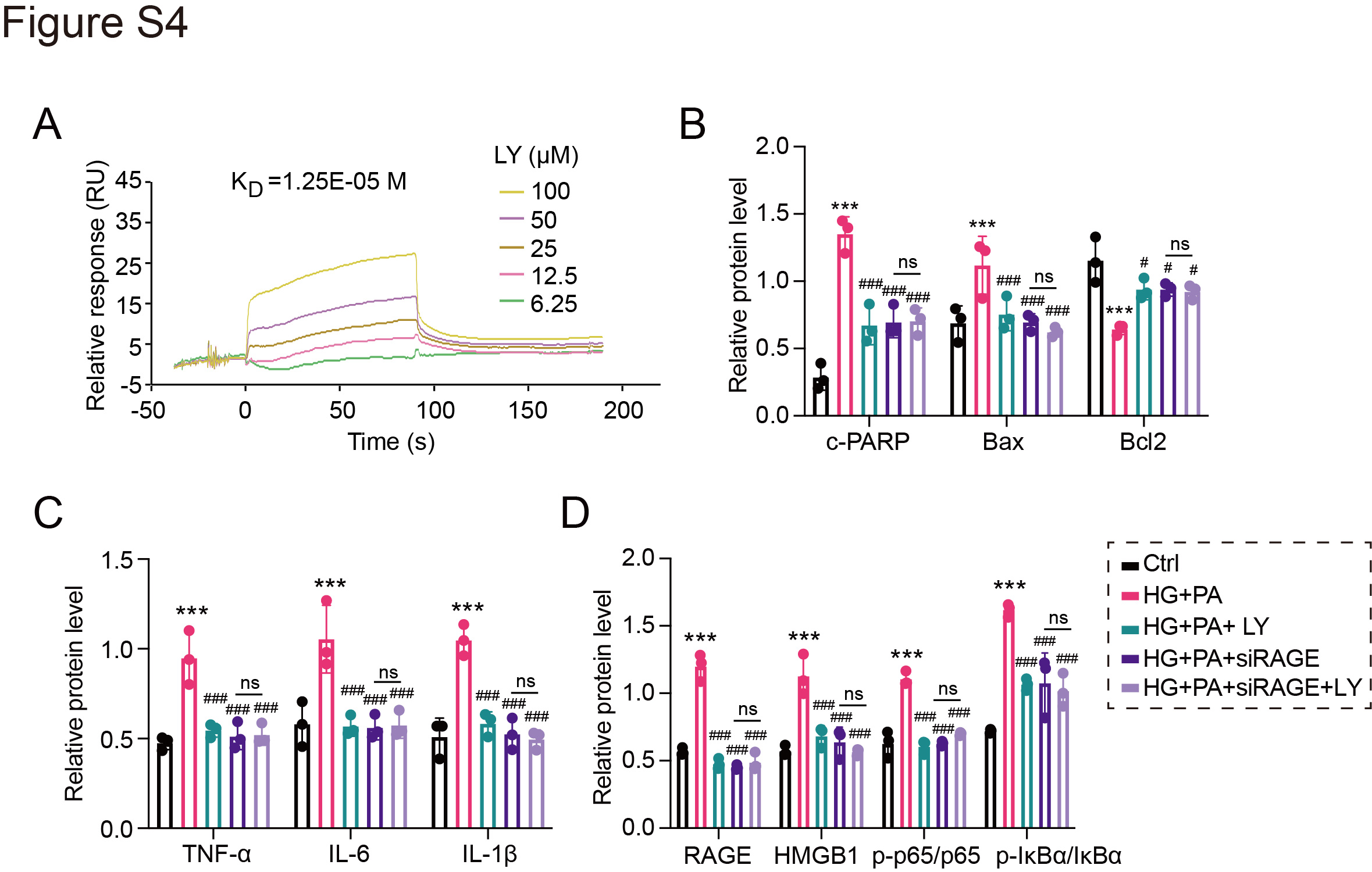

Supplement: Supplementary file 4 — Supplementary Material 4: Fig. S4. LY directly binds to RAGE and RAGE knockdown abolishes the additional protective effects of LY in HG + PA-treated cells.Surface plasmon resonanceanalysis showing the concentration-dependent binding of LY to recombinant RAGE protein. KD = 1.25 × 10⁻⁵ M.Quantitative analysis of apoptosis-related proteins, including cleaved PARP, Bax, and Bcl2, in control cells, HG + PA-treated cells, and cells subjected to LY treatment and/or RAGE knockdown.Quantitative analysis of inflammatory cytokine protein levels, including TNF-α, IL-6, and IL-1β.Quantitative analysis of HMGB1/RAGE/NF-κB signaling-related proteins, including RAGE, HMGB1, p-p65/p65, and p-IκBα/IκBα. Data are presented as mean ± SD. [file 13020_2026_1469_MOESM4_ESM.jpg]
